# Time‐series transcriptome reveals inflammatory signature in monocytes and neutrophils following acute heat exposure in mine rescuers

**DOI:** 10.14814/phy2.15946

**Published:** 2024-02-09

**Authors:** Jirui Wen, Ling Wang, Juan Cheng, Tengfei Ma, Qiao Wen, Can Li, Yuhao Zou, Xuehong Wan, Jiang Wu, Jifeng Liu

**Affiliations:** ^1^ Department of Otolaryngology Head and Neck Surgery/Deep Underground Space Medical Center, West China Hospital Sichuan University Chengdu China; ^2^ State Key Laboratory of Intelligent Construction and Healthy Operation and Maintenance of Deep Underground Engineering Sichuan University Chengdu China; ^3^ Med‐X Center for Manufacturing Sichuan University Chengdu China

**Keywords:** acute heat exposure, monocytes, neutrophils, transcriptome

## Abstract

Occupational exposure to extreme high temperatures and the increasing global temperatures necessitates a deeper understanding of the impact of heat exposure on human health. However, the molecular mechanisms underlying the response of monocytes and neutrophils to heat exposure in occupational population remain to be fully elucidated. This study used longitudinal transcriptome to assess the impact of acute heat exposure (50°C for 30 min) in 10 subjects from a mine rescue team before acute heat exposure (baseline) and at 5 min, 30 min, 1 h, and 24 h after acute heat exposure (recovery). The time‐series analysis revealed a coordinated molecular choreography of changes involving inflammation, coagulation, extracellular matrix, and energy metabolism. Importantly, the study characterized the inflammatory signature associated with heat exposure in monocytes and neutrophils, as evidenced by the rapid activation of the inflammation‐related transcriptome following heat exposure. Additionally, we pinpointed potential regulators, such as NR4A1, FOSL1, EGR3, and ATF3. In summary, the study suggested that the initial response to heat stress in monocytes and neutrophils from mine rescue team member was primarily characterized by a pro‐inflammatory stress response, which could potentially lead to the development of inflammation and ultimately result in a systemic inflammatory response in heatstroke.

## Graphical abstract

Time‐series transcriptome analysis of monocytes and neutrophils following acute heat exposure in mine rescuers. After acute heat exposure, transcripts could be clustered by different time longitudinal trajectories, reflecting a time‐dependent molecular responses. These molecular responses are related to inflammation, coagulation, extracellular matrix, and energy metabolism. Pro‐inflammatory stress response is the key feature in the initial phase of the response of monocytes and neutrophils to acute heat exposure, as evidenced by the rapid activation of the inflammation‐related transcriptome. Through transcription factor analysis, potential regulators of pro‐inflammatory stress response following acute heat exposure are identified, including NR4A1, FOSL1, EGR3, and ATF3.

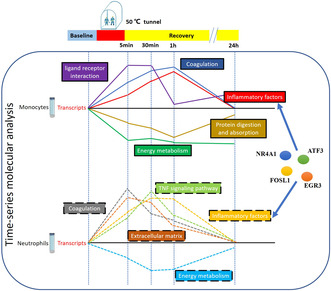

## 1 INTRODUCTION

Occupational exposure to extreme high temperature, as seen in industries such as steelmaking, tile production processes, mining, and rescuing, can lead to detrimental health effects (Lee et al., 2019). Especially, several studies showed that moisture and heat were the most commonly perceived adverse factors in the deep underground work environment (Liu et al., 2019; Strzemecka et al., 2019; Xie et al., 2020). Therefore, these studies have raised serious concerns about the impacts of extreme high temperature on occupational population health. Notably, individuals with occupational exposure in mining and rescuing experience high‐intensity physical activity in their daily lives, which may cause a certain degree of acclimation to heat exposure.

The previous mainly focused on describing the phenotypic and physiological responses associated with heat exposure. In deep mine, the miners maintained a core body temperature of 38°C for most of the day and experienced a significant increase in heart rate and severe dehydration because of the high‐ambient temperature (Lutz et al., 2014; Yeoman et al., 2019). These observations have raised the need to evaluate tissue and organ injury, monitor recovery, and predict long‐term complications following heat exposure. Notably, delayed measurement of core temperature or other physiological symptoms can result in unexpected occurrences of severe heat‐related illnesses, such as heatstroke, which can cause tissue damage and multiorgan dysfunction.

Monocytes are versatile cells capable of supporting tissue homeostasis, mediating host defense, and triggering inflammation, playing a crucial role in non‐infectious stress responses (Lin et al., 2023). Neutrophils, the most abundant immune cells, also play a central role in immune defense, contributing to various physiological functions including inflammation, angiogenesis, coagulation, and tissue repair (Liew & Kubes, 2019). Therefore, the activation state of monocytes and neutrophils is a critical factor in the body's response to stress. In a high‐temperature environment, monocytes and neutrophils undergo a series of heat stress changes. Luo et al. isolated and cultured human blood monocytes, and then subjected them to 43°C combined with lipopolysaccharide (LPS), 43°C alone, and LPS alone. The results showed that, compared to the control group, the expression of inflammatory mediators (TNF‐α, IL‐1β, IL‐10) and surface molecules (TREM‐1, TLR‐4, and CD86) in monocytes was significantly increased in the 43°C combined with LPS group, the 43°C group, and the LPS group, indicating that heat stimulation promotes the secretion of inflammatory mediators and phagocytosis in peripheral blood monocytes (Luo et al., 2019). Zhou et al. found that 42°C for 1 h can induce the expression of TLR2, TLR4, and HSP70 in the human monocyte cell line U937 cells and primary human monocytes, enhancing the immune response of monocytes (Zhou et al., 2005). Additionally, Liu et al. demonstrated that heat stress for 2 weeks in chickens induced liver infiltration of neutrophils and increased the expression of HSP70, NLRP3, caspase‐1, IL‐1β, IL‐6, TNF‐α, NF‐κB P65, and IκB in the livers, indicating activated inflammation and heat shock response (Liu et al., 2022). Given the varying tolerance of occupational populations to heat compared to the general population, it is likely that heat exposure may elicit distinct molecular reactions in these specific occupational groups. Thus, understanding the molecular responses in monocytes and neutrophils induced by extreme high temperatures can provide valuable insights into the physiological changes that occur under heat conditions. Monitoring these molecular responses can help us gain a better understanding of the occupational population's adaptive capacity to extreme high temperatures and assess the potential degree of damage. The objective of this study was to perform longitudinal transcriptome of blood components from 10 mine rescue team members, before and after a 30 min stay in a simulated extreme high‐temperature environment (50°C). The goal was to characterize the detailed series of events that occur in monocytes and neutrophils that response to acute heat exposure in occupational population. Findings revealed an orchestrated molecular choreography of changes main in inflammation reaction and provided the key transcription factors that regulated these processes.

## 2 METHODS

### 2.1 Study subjects

This study included 10 subjects recruited from the National Mine Emergency Rescue Furong Team (China) in May 2022. These team members usually undergo sports training and participate in coal mine rescue operations when disasters occur. The study protocol was approved by the Ethics Review Committee (IRB number, 1351) of West China Hospital of Sichuan University. All subjects provided written informed consent before participating in any study procedures. Subjects were screened for contraindications and/or comorbidities that may have prohibited exposing them to extreme high temperature using a health questionnaire. Subjects had no history of underlying cardiovascular disease, cancer, or other chronic diseases. No subject underwent heat exposure training within 3 months before the start of this study.

### 2.2 Study design

In a systematic review assessing the impact of occupational heat strain, the studies included in the analysis reported a wide range of WBGT (19.3–52.0°C) and air temperature (21.2–150.0°C, this extreme value was observed in a steel plant worksite) (Flouris et al., 2018). For a focused investigation to extreme high temperature exposure and ensures security considerations, we specifically chose 50°C as the environmental exposure temperature. The study was conducted in three phases: baseline, exposure, and recovery. The baseline period extended from 09:00 a.m. on the day before exposure until the day of exposure. The exposure period took place between 09:00 a.m. and 09:30 a.m. The recovery period extended from 09:30 a.m. until 09:00 a.m. the next morning. At 09:00 a.m. on the day of exposure, subjects were exposed to an extreme high temperature environment of 50°C and 37%–40% relative humidity for 30 min, using a mine roadway simulating test device designed to simulate fire and facilitate training of the National Mine Emergency Rescue Team. Heat was provided by industrial heaters (JH‐H150F, Cameron, China) placed at both ends of the roadway. During acute heat exposure, heart rate changes were monitored by an electronic sphygmomanometer. Immediately after the heat exposure, oral temperature was measured using a liquid crystal thermometer (WatermarkTM).

### 2.3 Blood collection and sample preparation

After an overnight fast, blood samples were collected from an upper forearm vein at baseline, 5 min, 30 min and 1 h after heat exposure, and the morning after heat exposure. Blood samples were collected in purple top vacutainers (BD), placed on ice, and processed immediately. Peripheral blood monocytes and neutrophils were isolated using a monocyte isolation kit (Solarbio, China, P8680) or neutrophil isolation kit (Solarbio, China, P9040), according to the manufacturer's instructions. Peripheral blood monocytes and neutrophils were divided into 4 equal portions and frozen at −80°C. Gene expression profiling was performed on monocytes and neutrophils (transcriptomics).

### 2.4 RNA sequencing and data processing

Total RNA was extracted using the TRIzol reagent (Invitrogen, USA, 15596–026) according to the manufacturer's protocol. Then the libraries were constructed using VAHTS Universal V6 RNA‐seq Library Prep Kit (Vazyme, China, NR604) according to the manufacturer's instructions. Pooled libraries were sequenced on a HiSeq 4000 sequencer (Illumina, San Diego, CA, USA) generating 30 million paired‐end (100 bp) reads per sample. Gene quantification was performed using the script htseq count in the Python software package HTSeq (v0.9.1). Genomic characteristics were defined using GENCODE v28, where each gene is considered a combination of all its exons. Gene counts were normalized for sequencing depth, and genes with a mean expression less than 10 were removed. Missing values were filled in with the k‐nearest neighbors' method using the “impute. knn” function in the R software package “impute” (v1.52.0). Two data sets were generated, one containing gene counts normalized for sequencing depth in each sample (raw counts), and the other was further processed with variance‐stable transformation (VST) using the R software package “DESeq2”.

### 2.5 Quantification and statistical analysis

#### 2.5.1 Differential expression analysis

We conducted differential expression analysis using the DESeq2 package. A q value <0.05 and a fold change >2 or fold change <0.5 were set as the threshold for significantly differential expression genes (DEGs). Hierarchical cluster analysis of the DEGs was performed using R (v 3.2.0) to illustrate the expression pattern of genes in different groups and samples. Additionally, we created a radar map of the top 30 genes to display the expression of up‐regulated or down‐regulated DEGs using the R package grader.

#### 2.5.2 Pathway enrichment analysis

KEGG and GO enrichment pathways analysis was used to identify differentially expressed plasma proteins and genes to determine enrichment of particular pathways. Hypergeometric probability (one‐sided) was used to determine the significance of each pathway. The Benjamini–Hochberg method was used to correct *p* values for multiple comparisons. FDRs of <0.05 and <0.20 were considered significant for transcripts. Fold change was estimated for significant molecules using (1) median of fold change relative to baseline, (2) median of beta coefficients, (3) median of maximum (if up) or minimum (if down) fold change relative to baseline.

#### 2.5.3 Transcription factors and their target gene prediction analysis

By comparing the distribution of transcription factors for all genes and differentially expressed genes (up‐regulated and down‐regulated), transcription factors with significant proportional differences can be identified. Further focused analysis can determine their association with differential expression. Differential target genes corresponding to differential transcription factors are extracted from the list of transcription factor and target gene relationships, and a chart of the target genes of the differential transcription factor family is generated.

#### 2.5.4 Fuzzy c‐mean clustering

After log2‐transformation and Z‐score scaling of data, fuzzy c‐mean clustering was conducted using the ‘Mfuzz’ R package (v2.20.0). The ‘elbow’ method was used to calculate the minimum centroid distance or a range of cluster numbers and to select the optimal number. t‐distributed stochastic neighbor embedding (tSNE) scatterplots were obtained using the ‘Rtsne’ R package, with the following parameters: perplexity = 5 and theta = 0.05.

### 2.6 qRT‐PCR

We performed quantitative real‐time PCR (qRT‐PCR) by reverse‐transcribing 2 μg RNA in a 50‐μL reaction using the high capacity cDNA reverse transcription kit, following the recommended instructions (Tiangen, China, KR116). Following the manufacturer's instructions, we conducted qRT‐PCR gene expression analysis using SYBR Green PCR Master Mix (Tiangen, China, FP207) for three replicates, selecting genes from the key transcription factors identified in the transcriptome results. We utilized GAPDH as the endogenous control for all gene expression analyses and performed the analysis with the Bio‐rad CFX96 real‐time PCR system. We used the 2^−ΔΔCt^ method to calculate the mean fold changes representing the expression level of a gene in each sample.

## 3 RESULTS

### 3.1 Cohort characteristics

**FIGURE 1:**
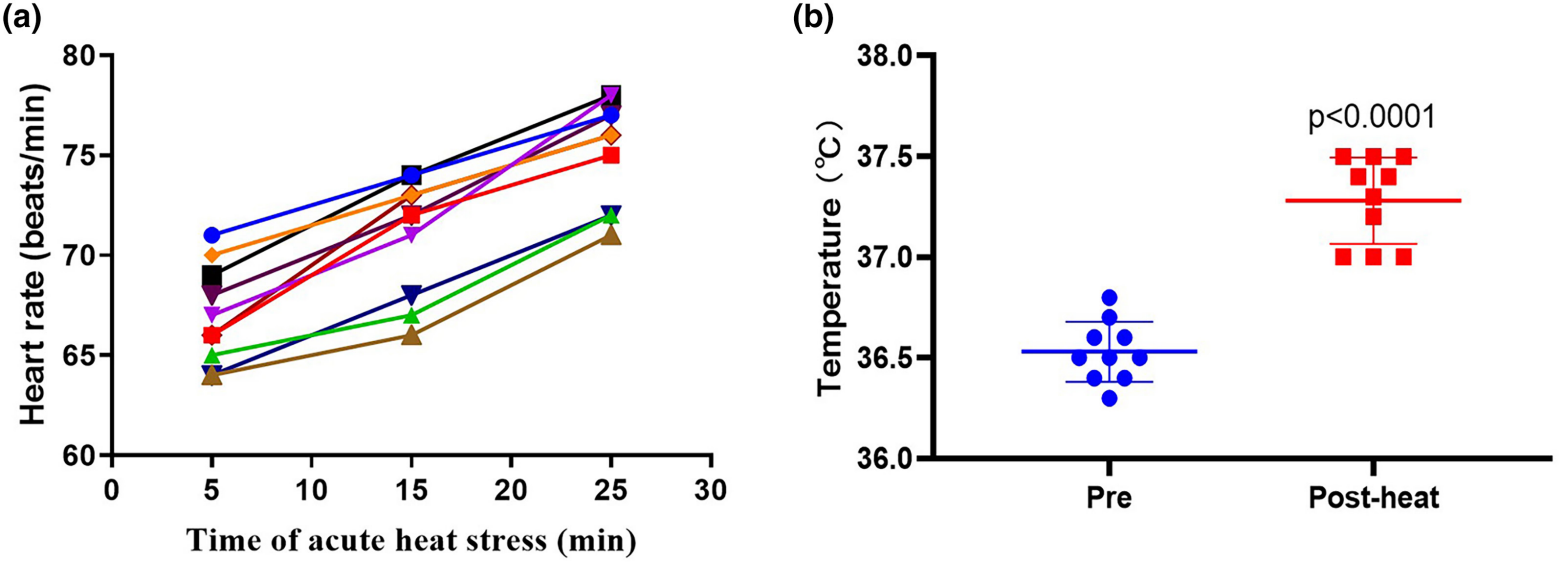
Heart rate and temperature changes in response to acute heat exposure. (a) Heart rate was detected during 30 min high temperature environment simulation training. (b) Temperature was detected before and after the acute heat exposure. Values are mean ± SD; mine rescuers, *n* = 10.

This study included 10 subjects. All were male, with a mean age of 32.4 years, mean height of 171.69 cm, and mean weight of 69.24 kg. Subjects were exposed to an extreme high temperature environment of 50°C and 37–40% relative humidity for 30 min. During acute heat exposure, there was an observed increase in heart rate (Figure 1a). Immediately after acute heat exposure, oral temperature rose to 37.3°C compared to 36.5°C at baseline (Figure 1b). These results suggested that subjects exhibit typical physiological responses to combat heat stress.

### 3.2 Gene expression signature

**FIGURE 2:**
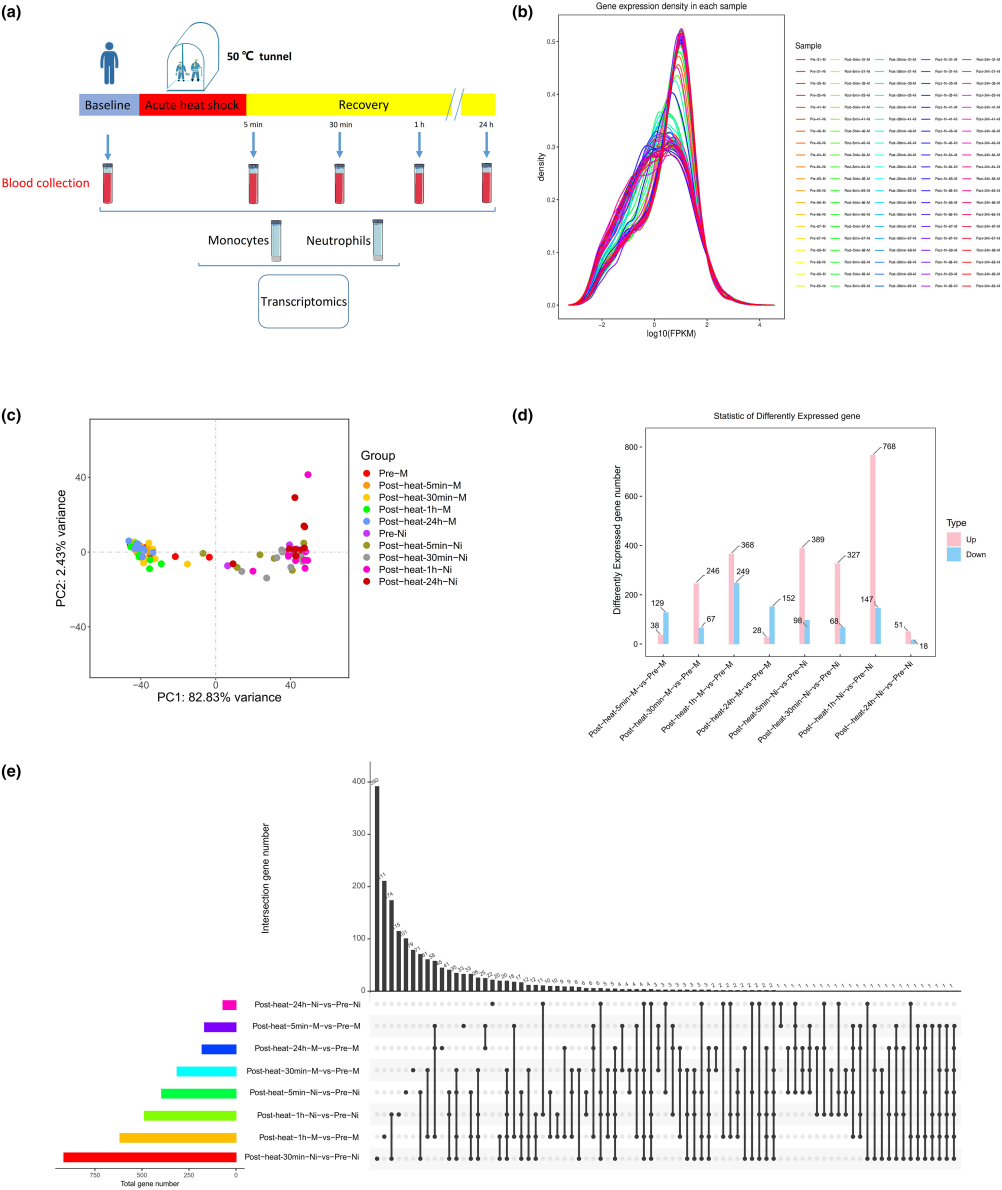
Study design and molecular response to acute heat exposure. (a) Overview of the study design. Subjects were exposed to an extremely high temperature environment of 50°C and 37%–40% relative humidity for 30 min. Subjects underwent a blood collection process in time series. (b) Density distribution curve. Different colored lines in the graph represent different samples, the *x*‐axis of the points on the curve represents the logarithmic value of the corresponding sample FPKM, and the *y*‐axis of the points represents the probability density. (c) Principal components analysis using transcriptomics data. (d) Statistical bar chart of the differentially expressed genes. The horizontal axis represents the different comparison groups and the vertical axis represents the number of differentially expressed genes in the comparison groups. (e) Common and unique differentially expressed genes among different comparison groups.

For the transcriptome analysis, intravenous blood samples were collected before acute heat exposure (baseline) and at 5 min, 30 min, 1 h, and 24 h after acute heat exposure (recovery) (Figure 2a). The FPKM density distribution reflected the expression patterns of genes in each sample, indicating normal gene expression patterns (Figure 2b). The omics datasets were assessed with principal component analysis, which suggested limited batch effects (Figure 2c). Exposure to an extreme high temperature environment of 50°C induced extensive changes in 3143 transcripts, indicating system‐wide changes. In monocytes, changes in the transcriptional profile peaked at 1 h after acute heat exposure. In neutrophils, changes in the transcriptional profile peaked at 30 min after acute heat exposure (Figure 2d). Figure 2e shows the number of commonly and specifically differentially expressed genes between different comparison groups, indicating that some genes were differentially expressed in both monocytes and neutrophils.

### 3.3 Differentially expressed genes

**FIGURE 3:**
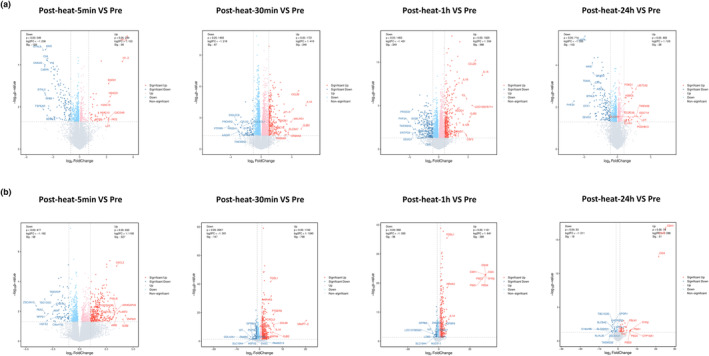
Differentially expressed genes in the peripheral monocytes and neutrophils at different time points. (a) Differential expression of the comparison in the monocytes is reflected in the volcano plot, where gray represents nonsignificantly different genes, and red and blue represent significantly different genes. (b) Differential expression of the comparison in the neutrophils is reflected in the volcano plot.

**FIGURE 4:**
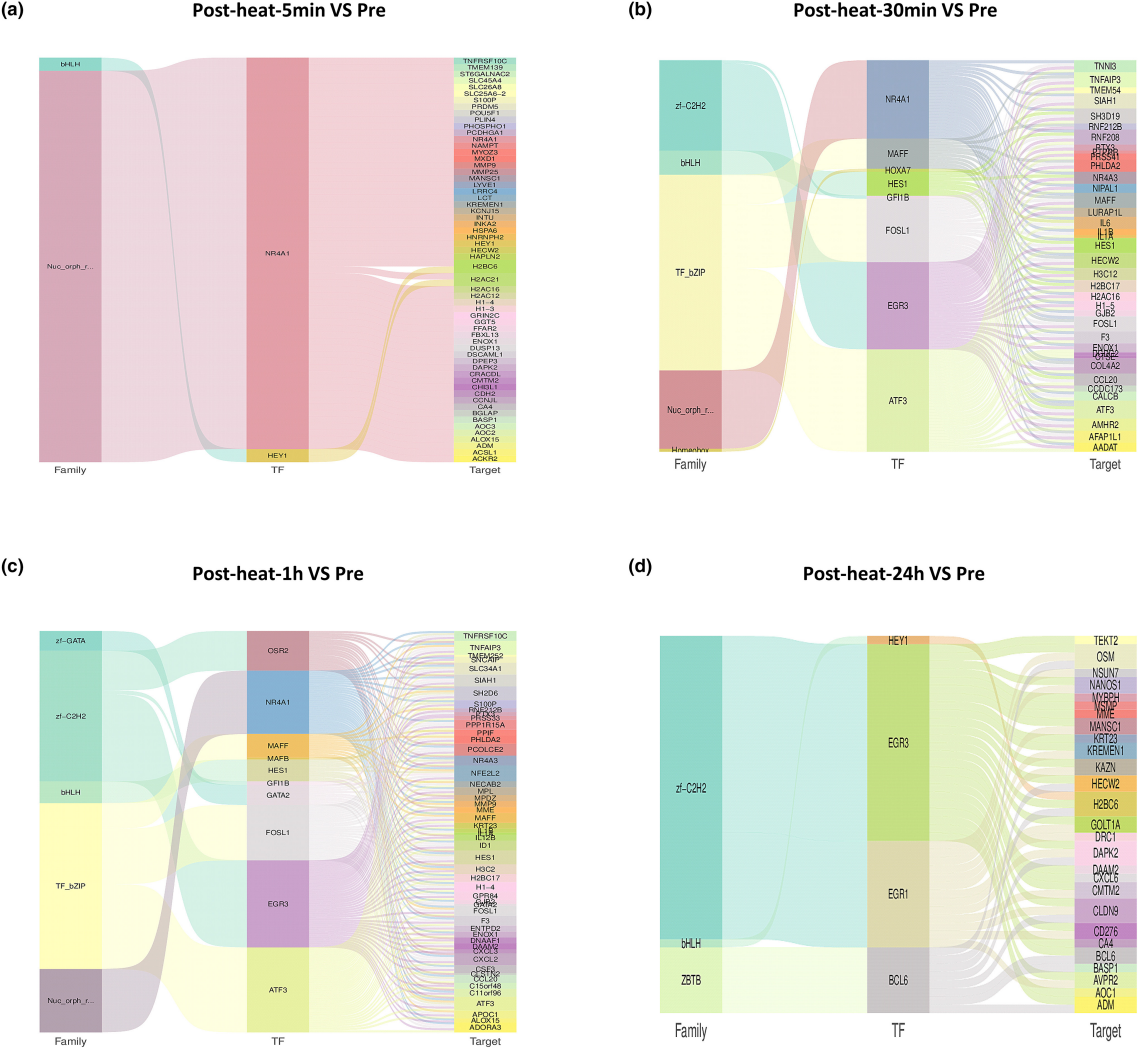
Transcription factor analysis in the peripheral monocytes at different time points. From left to right: the first column represents the transcription factor family, the second column represents the differential transcription factors, and the third column represents the differential target genes. The lines in the middle represent the corresponding relationships between the transcription factor family, the transcription factor, and the target gene.

**FIGURE 5:**
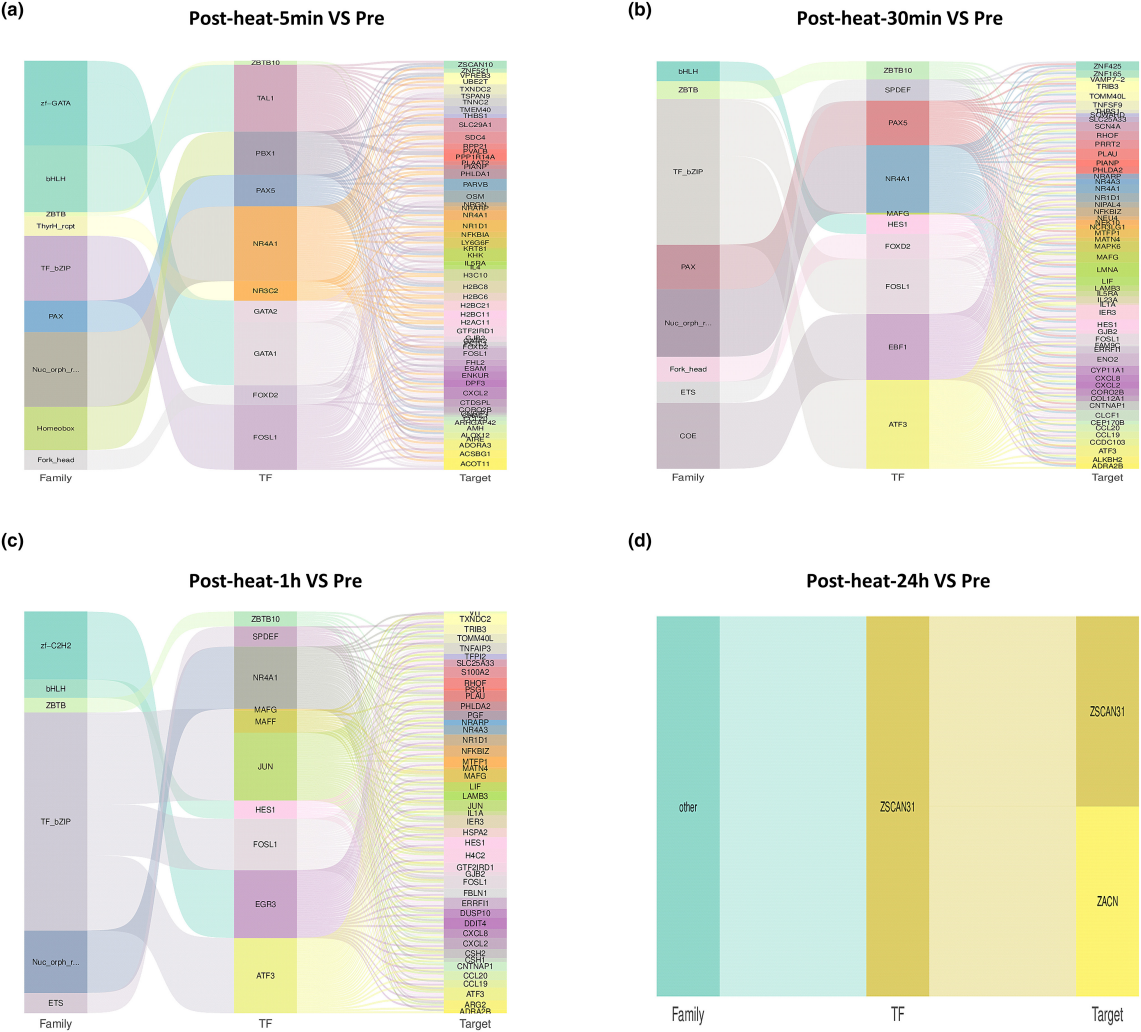
Transcription factor analysis in the peripheral neutrophils at different time points. From left to right: the first column represents the transcription factor family, the second column represents the differential transcription factors, and the third column represents the differential target genes. The lines in the middle represent the corresponding relationships between the transcription factor family, the transcription factor, and the target gene.

Volcano graphs of differentially expressed genes between different comparison groups showed that various chemokines and inflammatory factors related genes were the genes most expressed with the highest fold changes (Figure 3). Through transcription factors analysis, we identified transcription factors exhibiting expression changes at various time points (Figures 4 and 5). Among these, NR4A1, FOSL1, EGR3, and ATF3 were found to regulate a greater number of target genes and were associated with more inflammatory‐related genes. Notably, the expression of these four transcription factors showed changes in both cell types and at multiple time points, indicating sustained alterations following heat exposure.

### 3.4 Time‐series analysis of the transcriptomic profile of peripheral blood monocytes

**FIGURE 6:**
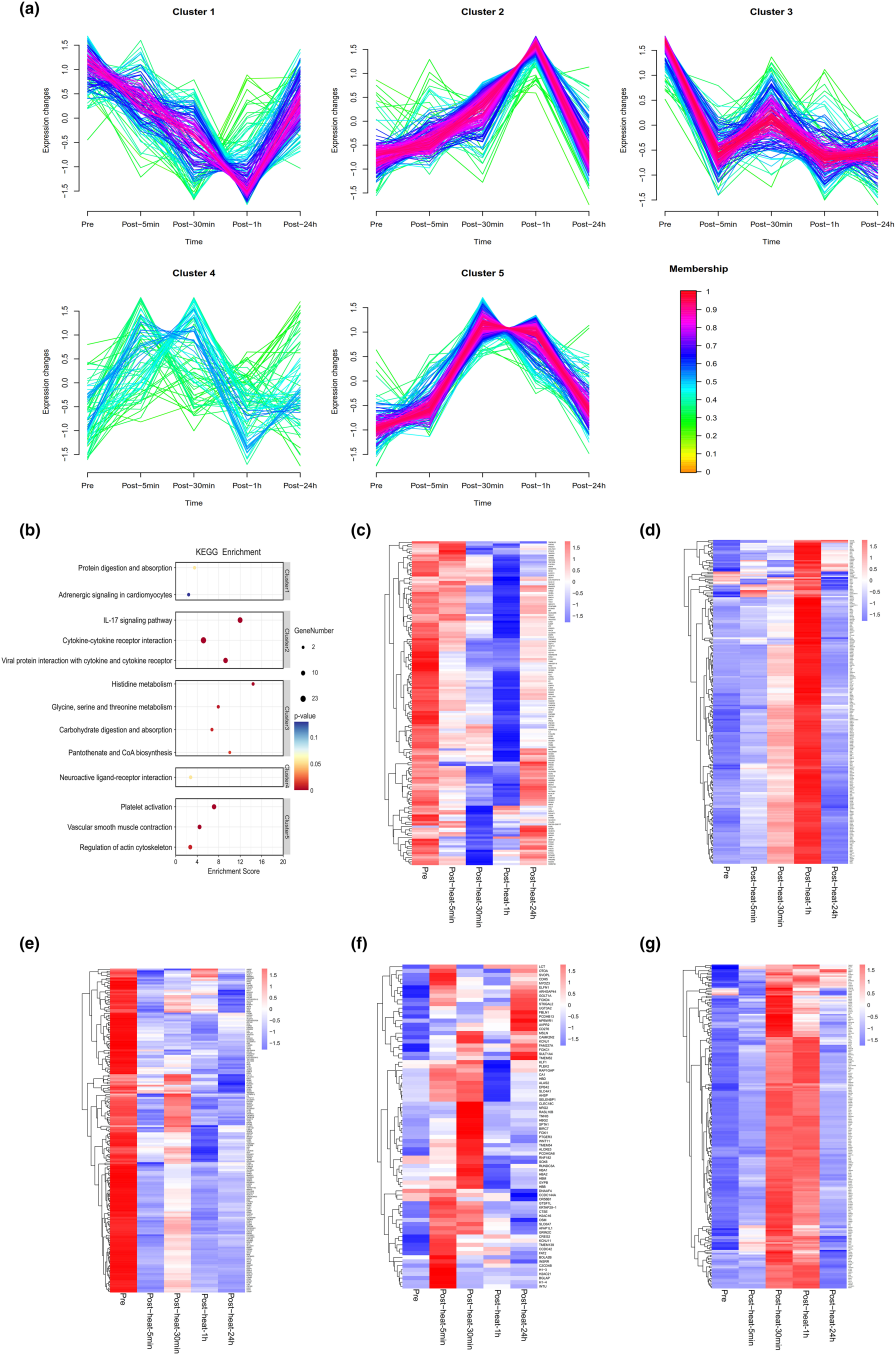
Time‐series analysis of the transcriptomic profile of peripheral blood monocytes. (a) Clustering of longitudinal trajectories using significant circulating monocyte transcripts. (b) Pathway enrichment analysis. Pathway direction is the median log2 fold change relative to baseline of significant molecules in each pathway (blue, downregulated; red, upregulated). Dot size represents pathway significance. (c–g) Heatmaps representing longitudinal trajectories of monocyte transcripts in cluster 1 (c), cluster 2 (d), cluster 3 (e), cluster 4 (f), and cluster 5 (g). Gene names are on the right (blue, downregulated; red, upregulated).

Cluster analysis (k‐means clustering) identified five clusters of transcriptomic profile of peripheral blood monocytes with different longitudinal trajectories. The levels of some transcripts decreased after acute heat exposure and returned to baseline within 24 h (cluster 1). The levels of some transcripts decreased after acute heat exposure and did not return to baseline within 24 h (cluster 3). The levels of some transcripts increased after acute heat exposure, reached a maximum response at different time points, and returned to baseline within 24 h (cluster 2, cluster 4, cluster 5) (Figure 6a).

Pathway enrichment analysis was performed on each cluster (Figure S1). Cluster 1 was enriched in protein digestion and absorption; cluster 2 was enriched in cytokine–cytokine receptor interaction; cluster 3 was enriched in glycine, serine, and threonine metabolism; cluster 4 was enriched in neuroactive ligand–receptor interaction; and cluster 5 was enriched in platelet activation (Figure 6b). Representative genes in each cluster are shown in Figure 6c–g and Table S1.

### 3.5 Time‐series analysis of the transcriptomic profile of peripheral blood neutrophils

**FIGURE 7:**
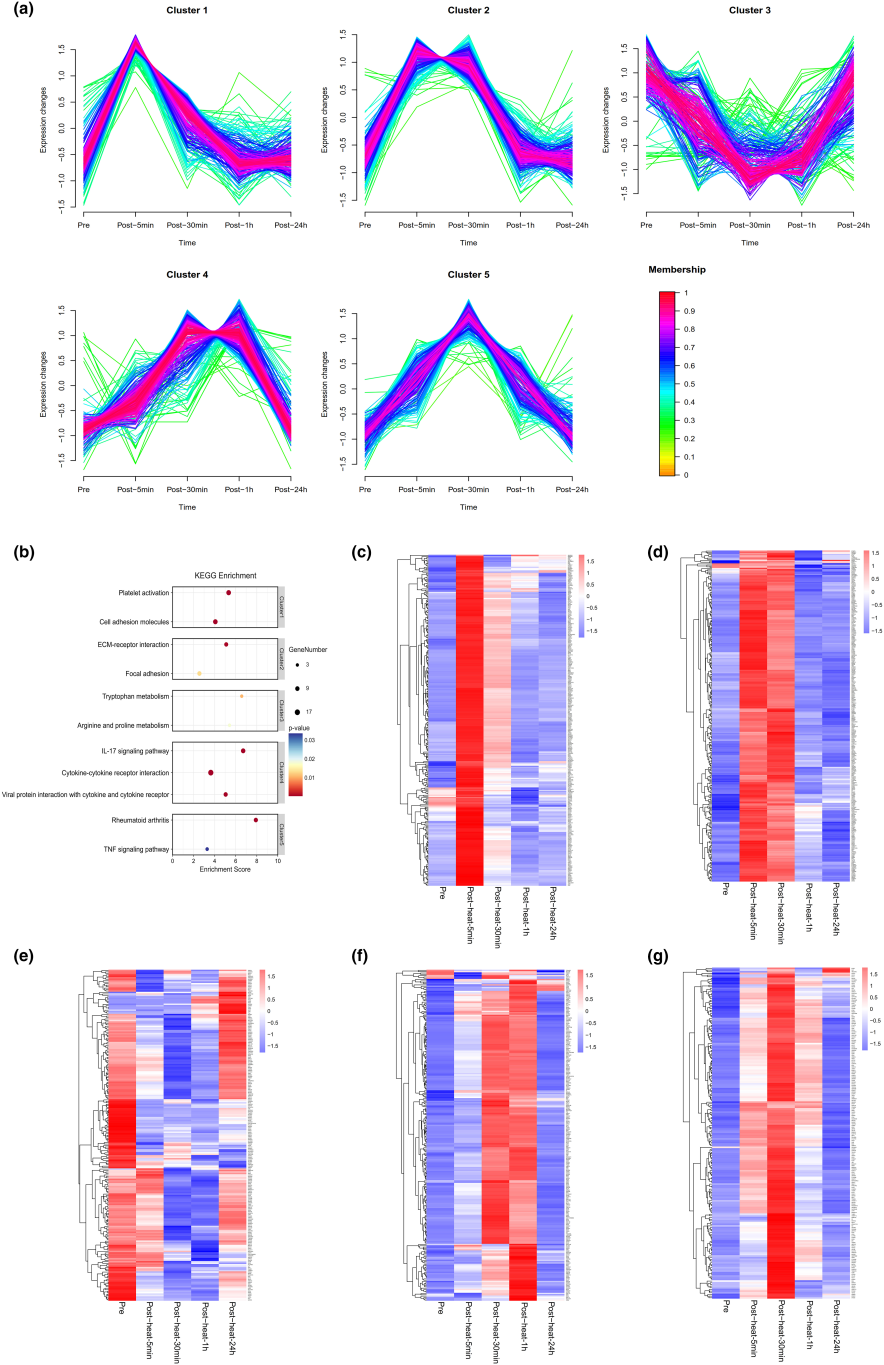
Time‐series analysis of the transcriptomic profile of peripheral blood neutrophils. (a) Clustering of longitudinal trajectories using significant circulating monocyte transcripts. (b) Pathway enrichment analysis. Pathway direction is the median log2 fold change relative to baseline of significant molecules in each pathway (blue, downregulated; red, upregulated). Dot size represents pathway significance. (c–g) Heatmaps representing longitudinal trajectories of neutrophil transcripts in cluster 1 (c), cluster 2 (d), cluster 3 (e), cluster 4 (f), and cluster 5 (g). Gene names are on the right (blue, downregulated; red, upregulated).

Transcript trajectories in peripheral blood neutrophils were categorized into five clusters. The levels of some transcripts increased after acute heat exposure reached a maximum response at different time points, and returned to baseline within 24 h (cluster 1, cluster 2, cluster 4, cluster 5). The levels of some transcripts decreased after heat exposure and returned to baseline within 24 h (cluster 3) (Figure 7a).

Pathway enrichment analysis was performed for each cluster (Figure S2). Cluster 1 was enriched in platelet activation. Cluster 2 was enriched in ECM‐receptor activation. Cluster 3 was enriched in tryptophan metabolism. Cluster 4 was enriched in cytokine–cytokine receptor interaction. Cluster 5 was enriched in the TNF signaling pathway (Figure 7b). Representative genes in each cluster are shown in Figure 7c–g and Table S1.

### 3.6 Validation of the transcriptome

**FIGURE 8:**
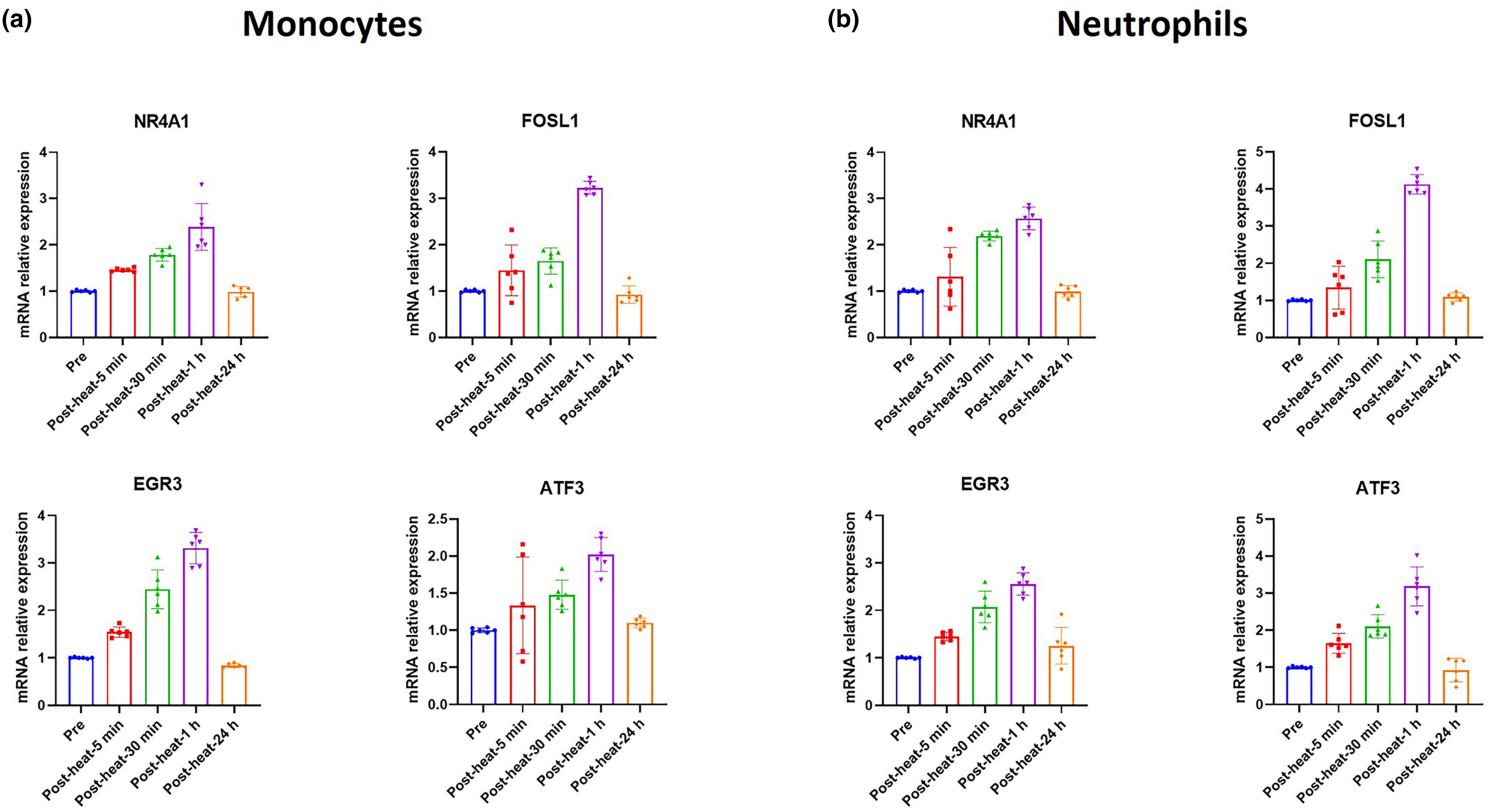
Validation of the representative transcription factors. NR4A1, FOSL1, EGR3, and ATF3 levels before and after acute heat exposure were detected by RT‐qPCR. GAPDH was utilized as the endogenous control for all gene expression analyses and the 2^−ΔΔCt^ method was used to calculate the mean fold changes representing the expression level of a gene in each sample. Values are mean ± SD; mine rescuers, *n* = 6.

Among transcription factors, NR4A1, FOSL1, EGR3, and ATF3 were found to show changes in both cell types and at multiple time points. The time‐series analysis also indicated that these four transcription factors exhibited similar temporal trends to inflammation‐related genes, which continued to increase after acute heat exposure and returned to baseline the next day. Thus, we selected these four representative transcription factors from transcriptome to validate. We found a significant concordance between the transcriptome and the RT‐qPCR data in both monocytes and neutrophils (Figure 8).

## 4 DISCUSSION

Occupational exposure to extreme high temperatures and increasing global temperatures require enhanced understanding of how heat exposure impacts human health (Ebi et al., 2021). Monocytes and neutrophils are now recognized as functionally flexible. This study characterized the detailed series of events that occur in monocytes and neutrophils that respond to acute heat exposure (50°C). We uncovered a specific inflammation signature of monocytes and neutrophils, reflecting the exposure to environmental signals. Integrated control of inducible transcriptional programs was related to elicitation of stimulus‐specific functional responses, which revealed acute heat exposure induced a time‐dependent molecular responses. These molecular responses were related to the immune response, coagulation, extracellular matrix, and energy metabolism, which provided insight into the potential physiological alterations in response to acute heat exposure. Time‐series data showed variations in the dynamics of these molecules, shedding light on the diverse temporal patterns of molecular changes in response to acute heat exposure. NR4A1, FOSL1, EGR3, and ATF3 were identified as potential regulators of the chemokines and inflammatory factors involved in the response to acute heat exposure.

Compared with monocytes, the changes in the transcriptional profile of neutrophils were more and faster, which peaked at 30 min after acute heat exposure. These results suggested rapid mobilization of neutrophils. Notable, there were some commonly differentially expressed genes in both monocytes and neutrophils. These genes may reflect the common reactions of immune cells when exposed to an extreme high temperature environment of 50°C, which was the focus of this study.

The present study showed that acute heat exposure induced proinflammatory cytokines, and monocytes and neutrophils may mediate this response. The transcriptome of peripheral blood monocytes exposed to acute heat exposure had an inflammatory signature consisting of chemokines (C‐C motif ligand, C‐X‐C motif ligand, interleukin), complement C1qA chain, IFN‐γ, TNF, NLRP3, and colony‐stimulating factor 3. Accumulating evidence indicates that multiple inflammatory cytokines such as TNF‐α, IFN‐γ, CCL20, IL1, and IL6 induce inflammation (Dinarello, 2011; Hanna et al., 2022; Jones & Jenkins, 2018; Karki et al., 2021). C1qA, the initiating molecule of the classical complement cascade, can trigger inflammation, and blocking C1q function reduces chronic glial inflammation and neuron loss (Holden et al., 2021). NLRP3‐inflammasome plays a role in inflammatory signaling (Anzai et al., 2021; Karasawa & Takahashi, 2017). CSF3 regulates granulocytes and monocytes and has inflammatory properties (Dougan et al., 2019). Notably, the timing and magnitude of heat exposure may determine the type of inflammatory response. In our study, with short‐term heat exposure, the inflammatory transcriptome can return to baseline levels. However, long‐term and high‐intensity heat exposure may induced sustained activation of the host inflammatory response, which may explain the “systemic inflammatory response syndrome” and multiple organ failure in severe heat‐related illness. Anti‐inflammatory agents targeting factors involved in the inflammatory response to heat exposure may be beneficial at early time after heat exposure, with potential to prevent progression of heat‐related illnesses. This finding also raised the question of what was the fundamental mechanism that induced the inflammatory signature.

This study offers several potential regulators for this mechanism. Through transcription factors analysis, we identified some transcription factors (NR4A1, FOSL1, EGR3, ATF3) may regulate the multiple inflammation‐related genes. These four genes, NR4A1, FOSL1, EGR3, and ATF3, had been shown to be closely associated with inflammation in previous studies. For example, the activation of NR4a1/Nur77 improved hepatic inflammation and fibrosis in hepatic stellate cells (Fuchs et al., 2023). Fos‐like antigen 1 (FOSL1), a member of the Fos family, had been implicated in the activation of inflammatory pathways in various cell types, and FOSL1 knockdown was demonstrated to ameliorate inflammatory injury (Ma et al., 2022). Another study proved that Early Growth Response 3 (EGR3) regulated the expression of genes involved in immune responses and inflammatory processes, and directly activated IL6 and IL8 expression (Baron et al., 2015). Activating transcription factor‐3 (Atf3) also contributed to inflammation, which had promoting effects in particulate matter‐induced airway inflammation in vitro and in vivo (Willemsen et al., 2022; Yan et al., 2018). Interestingly, the time‐series analysis indicated that NR4A1, FOSL1, EGR3, and ATF3 exhibited similar temporal trends to inflammation‐related genes, which continued to increase after acute heat exposure and returned to baseline the next day, implying these genes were potential regulators of the inflammatory processes involved in the response to acute heat exposure.

Abnormal coagulation was a common feature of heat stroke, and the severe disseminated intravascular coagulation (DIC) in heat stroke may cause the fatal outcome (al‐Mashhadani et al., 1994; Bouchama et al., 1996; Jilma & Derhaschnig, 2012). In our study, we observed an increase in transcripts related to platelet activation in response to acute heat exposure, highlighting the communication function of monocytes and neutrophils. These elevated coagulation‐related factors reflect a hypercoagulant state following acute heat exposure, potentially contributing to the development of coagulation dysfunction in individuals exposed to high temperatures. The activation of monocytes and neutrophils may serve as risk factors for coagulation dysfunction and heat stroke‐related DIC.

Following acute heat exposure, we observed a decrease in genes related to amino acid metabolism in monocytes and neutrophils, suggesting that high temperature environments may adversely affect cellular protein synthesis processes. Additionally, downregulation of genes related to protein and carbohydrate digestion and absorption may indicate reduced nutrient utilization. Overall, the decrease in energy production may partially underlie the pathogenic mechanisms contributing to impaired cellular function and survival. Notably, in neutrophils, we observed an increase in genes related to the extracellular matrix. As previously reported, neutrophils can mediate ECM remodeling, generating neutrophil extracellular traps, and releasing exosomes (Zhu et al., 2021). Thus, the interplay between the ECM and neutrophils may represent an important aspect of acute heat stress.

This study had some limitations, including a small sample size that did not include females or the elderly. Additionally, the maximum tolerance time was not detected due to safety concerns for subjects. As a result, molecular changes associated with life‐threatening heatstroke were not explored. In a notable study by Bouchama et al., the whole genome transcriptome in peripheral blood mononuclear cells of an adult cohort with heatstroke was examined. It was found that in heatstroke, the heat shock response was robust but failed to restore homeostasis due to proteotoxicity and a reduction in energy production. This study suggested that the heat shock response and severe cell damage were the main physiological processes in severe heat‐related diseases. However, the variation in heat shock proteins under heat exposure largely depends on the magnitude of temperature increase and the duration of heat exposure. Hoekstra et al. (2019) found that at lower temperatures (38.5°C), the impact on HSP72 in peripheral blood monocytes was minimal; at 40.0°C, the expression of endogenous HSP72 significantly increased. Lee et al. (2015) discovered that after heat acclimation (3 days), the response of monocyte HSP72 to hypoxic exercise diminished, indicating that an increased basal reserve of HSP72 can mediate improved heat tolerance and the ability to cope with hypoxic injury. In our study, no significant heat shock response was found, but a transcriptomic feature with inflammatory signals was discovered, indicating that the pro‐inflammatory stress response was the main feature in the early heat stress of monocytes and neutrophils. This could be attributed to the short‐term heat exposure and insufficient heat intensity to induce heat shock reactions. Alternatively, it may be due to the acclimatization of mine emergency rescue personnel to heat. This response of monocytes and neutrophils may partially explain the occurrence of systemic inflammatory response in heatstroke, as the inflammation‐related transcriptome was quickly activated after heat stress. Therefore, our research also has significant implications. This suggested that when the heat shock response is not yet activated, the inflammatory signal may be triggered first, posing a potential risk to the mine rescue team member.

## 5 CONCLUSION

Overall, this study offered insights into key research gaps in the health impact assessment of heat exposure in occupational population. Longitudinal transcriptome profiling identified thousands of molecules that were affected by acute heat exposure, and time‐series clustering revealed the changes in inflammation, coagulation, metabolism and extracellular matrix. Most importantly, we characterized the inflammatory signature associated with heat exposure in monocytes and neutrophils, and identified the potential regulators including NR4A1, FOSL1, EGR3, and ATF3. It helped to recognize the possible pathogenic molecular mechanisms that contributed to the severe heat injury. These findings have significant implications for occupational population exposed to heat, which may uncover clinically relevant biomarkers and support the development of diagnostic and therapeutic tools for heat‐related diseases.

## AUTHOR CONTRIBUTIONS

Jirui Wen, Jiang Wu, and Jifeng Liu contributed to the conceptualization. Jirui Wen, Ling Wang, and Juan Cheng contributed to the omic data generation and/or processing. Tengfei Ma and Qiao Wen contributed to recruitment of subjects and measurement of clinical parameters. Can Li and Yuhao Zou contributed to the data curation and data visualization. Xuehong Wan contributed to project administration and supervision. Jirui Wen, Ling Wang, Juan Cheng, Jiang Wu, and Jifeng Liu contributed to writing and preparing the original draft.

## FUNDING INFORMATION

We would like to express their gratitude for the support provided by 1.3.5 project for disciplines of excellence, West China Hospital, Sichuan University (ZYJC21048), Foundation of Sichuan Provincial Science and Technology Program (2022JDR0091, 2023NSFSC0004, 2023NSFSC0639, 2023NSFSC1742, 2021YFS0134), 2020 Cooperation project for Sichuan University and Yibin Municipal People's Government (2020CDYB‐35), Cooperation Project for Academician and Expert Workstation (HXYS20001), Sichuan University Education Foundation (0040206107011), and National Natural Science Foundation of China (82371883).

## CONFLICT OF INTEREST STATEMENT

The authors declare that they have no actual or potential competing financial interests.

## ETHICS STATEMENT

The study protocol was approved by the West China Hospital Ethics Committee, and all methods were conducted in strict adherence to the relevant guidelines and the Code of Ethics of the World Medical Association (Declaration of Helsinki). Prior to participation, the study subjects provided written informed consent.

## Supporting information


**Figure S1:** The Monocyte Gene Pathway Enrichment Analysis in Response to Acute Heat Exposure.
(A) Go enrichment analysis of five different clusters of Monocyte transcript.
(B) KEGG enrichment analysis of five different clusters of Monocyte transcript. The dot color changes from blue to red, representing a significant meaning. The dot size represented the gene number enriched in pathway.

**Figure S2:** The Neutrophils Gene Pathway Enrichment Analysis in Response to Acute Heat Exposure.
(A) Go enrichment analysis of five different clusters of Neutrophils transcript.
(B) KEGG enrichment analysis of five different clusters of Neutrophils transcript. The dot color changes from blue to red, representing a significant meaning. The dot size represented the gene number enriched in pathway.

**Table S1:** Marker genes of monocytes and neutrophils in different clusters defined by longitudinal trajectories.

## Data Availability

The transcriptomics data have been deposited in the GEO with the dataset identifier SUB13722246. The other datasets are available from the corresponding author upon reasonable request.
