# Supplementary material for: Time‐series transcriptome reveals inflammatory signature in monocytes and neutrophils following acute heat exposure in mine rescuers
Source: Physiol Rep. 2024 Feb 9;12(3):e15946. doi: 10.14814/phy2.15946 (PMC10858336; doi:10.14814/phy2.15946)
Supplement: Supplementary file 3 — Table S1: Marker genes of monocytes and neutrophils in different clusters defined by longitudinal trajectories. [file PHY2-12-e15946-s001.docx]

**Table 1. Marker genes of monocytes and neutrophils in different clusters defined by longitudinal trajectories**

| Monocytes | | | Neutrophils | | |
| --- | --- | --- | --- | --- | --- |
| Cluster | Transcripts | Gene | Cluster | Transcripts | Gene |
| Cluster 1 | Protein digestion and absorption | ITGB8, CTNNA2, KCNE1B, CACNG8, CACNB2 | Cluster 1 | Platelet activation | GUCY1A1, P2RY12, MYLK, GUCY1B1, ITGA2B, F2RL3, ARHGEF12, GP5, GP1BB, GP9, P2RY1, PRKG1 |
|  | Cardiovascular function | COL17A1, CPA3, COL15A1 |  | Cell adhesion | JAM3, CD226, HLA-DPA1, CNTNAP2, HLA-DQA1, CTLA4, ICOS, PDCD1, ESAM, NEO1, CNTNAP1 |
|  | Arginine and proline metabolism | ARG1, P4HA2 |  |  |  |
| Cluster 2 | Chemokines and inflammatory factors | C1QA, CCL20, CCL3, CCL3L3, CXCL1, CXCL2, CXCL3, CXCL8, IFNG, IL12B, IL1A, IL1B, IL23A, IL6, JUN, TGFB2, TNF; DYNLRB2, NR4A1, HBEGF, MAPK10, NFKBIA, NLRP3, RIPK2, ARG2, CSF3, ICAM1, IDO2, IFNG, LAMB3, PPIF, PTGS2, THBS1, CCL4, CCL4L2, CDKN1A, CXCL10, EGR3, HES1, HES4, PDGFRA, PTGS2, TNFAIP3, ZFP36; CXCL9, ATF3, FOSL1, KITLG, MARCKS, NFKBIE, P2RY12, PF4V1, PTGS2, RAPGEF4, SERPINB2 | Cluster 2 | Focal adhesion | ITGB3, LAMA5, AKT3, CAV2, ITGB8, COL6A3, BIRC3, FLT4 |
|  |  |  |  | ECM-receptor interaction | COL19A1, ATP1B1, CPA3, COL6A3, SLC8A3, COL24A1 |
| Cluster 3 | Amino acid metabolism | AOC1, HAL, HDC, AOC3, AOC2, CBS | Cluster 3 | Tryptophan metabolism | MAOA, IDO1, CYP1A1 |
|  | Carbohydrate digestion and absorption | MGAM, MGAM2, ATP1B2 |  | Arginine and proline metabolism | AMD1, MAOA, P4HA2 |
|  | Pantothenate and coa biosynthesis | VNN3, VNN2 |  |  |  |
| Cluster 4 | Histone subunit related transcripts | H2AC16, H2AC21 | Cluster 4 | Chemokines and inflammatory factors | CCL3L3, CXCL1, CXCL6, CXCL8, ICAM1, NR4A1, TNF, FCAR, HBEGF, HSPA2, IL1A, IL1B, JUN, NFKBIA, TUBB2A, ARG2, LAMB3, PPIF, CCL4, CCL4L2, COL1A1, EGR2, EGR3, FOSL1, FZD5, FZD7, GADD45B, HES1, HIF1A, JAG1, ATF3, NOTCH4, NXT1, TNFAIP3, YWHAG, ZFP36; CCL19, DLL4, FOSB, NFKBIE, PLAU, PLAUR, TFRC |
|  | Active ligand receptor interaction related transcripts | GRIN2C, PTGER3, AVPR2, NPBWR1 |  |  |  |
| Cluster 5 | Platelet activation | GP1BB, ITGA2B, MYLK, PTGS1, GP1BA, GP6, TBXA2R, GP9, ITGB3, VWF, F2RL3, ADCY5 | Cluster 5 | TNF signaling pathway | CXCL3, IL6, CCL20, CXCL2, TNFSF11, CFL2, DEFA3, DEFA4, IFNG, IL23A, SOCS1, THBS1, CCL3 |
|  | Vascular smooth muscle contraction | MYLK, CALD1, PPP1R14A, AVPR1A, RAMP1, CALCB, MYL9, ADCY5 |  |  |  |
|  | Cytoskeleton | GRIN2C, PTGER3, AVPR2, NPBWR1 |  |  |  |
|  | Complement and coagulation cascade | CLU, PROS1, VWF, F2RL3, PLAU and TFPI |  |  |  |
